# SpikeAEC: a neuromodulation-based spiking controller for explore-exploit balancing in mobile robots

**DOI:** 10.3389/fnbot.2026.1757795

**Published:** 2026-03-05

**Authors:** Canyang Liu, Yichen Liu, Yongqi Zhou, Buqin Su

**Affiliations:** 1 School of Artificial Intelligence, Nanjing University of Information Science and Technology, Nanjing, China; 2 School of Electronics and Information Engineering, Nanjing University of Information Science and Technology, Nanjing, China; 3 School of Computing, Nanjing University of Information Science and Technology, Nanjing, China

**Keywords:** actor-explorer-critic, exploration-exploitation dilemma, neuromodulation, spiking neural networks (SNNs), three-factor learning rules

## Abstract

Balancing exploration and exploitation remains a fundamental challenge in reliable mobile robot control, as conventional policies often converge on suboptimal behaviors. Inspired by the brain's division of labor for adaptive control, we propose SpikeAEC, a fully spiking, neuromodulated Actor-Explorer-Critic architecture designed to address this dilemma online within a closed-loop system. SpikeAEC comprises three specialized subnetworks operating in parallel: the Actor, inspired by the basal ganglia, proposes exploitative actions; the Explorer, modeled after the ACC-GPe-STN pathway, generates adaptive exploratory actions gated by a vigilance signal modulated by the accumulated global temporal-difference (TD) error; and the Critic, based on the ventral striatum, computes the TD error. The final action is selected by a separate, TAN-based Arbitrator, which probabilistically chooses between the Actor's and Explorer's action proposals according to recent performance and the TD error. These subnetworks are coupled through a unified three-factor learning framework that uses the TD signal and phasic neuromodulators (acetylcholine and dopamine) from the Arbitrator to drive pathway-specific synaptic plasticity. This online plasticity enhances the quality of action proposals and accelerates policy refinement. In simulation, SpikeAEC outperforms leading brain-inspired methods by converging 24% faster, reducing trajectory length by 18%, and increasing cumulative reward by over 5% against the top-performing baseline, all while maintaining consistency with established neurophysiological principles.

## 1 Introduction

Collision-free motion planning represents a central challenge in robotics, particularly for autonomous agents operating within dynamic and cluttered environments. The emergence of event-based cameras and neuromorphic processors, capable of delivering sub-millisecond response times, has significantly expanded the possibilities for robotic agility, especially in scenarios that require rapid navigation through constrained spaces (Paredes-Vallés et al., 2024). However, the availability of such unprecedented sensory-motor speed alone does not ensure reliable decision-making or safe trajectory planning. Effective control in highly dynamic contexts demands a sophisticated mechanism that balances exploitation, selecting actions currently estimated to be optimal, with exploration, evaluating alternative actions that may yield superior long-term outcomes (Dong et al., 2024). Striking this balance remains a critical and unresolved problem, as many existing control frameworks, ranging from classical motion planners to contemporary deep learning approaches, continue to struggle with robust and efficient online adaptation (Vashisth et al., 2024).

In response to these challenges, Spiking Neural Networks (SNNs) have emerged as a compelling computational paradigm for real-time control. Unlike conventional Artificial Neural Networks (ANNs), SNNs process information through discrete, asynchronous spike events, enabling sub-millisecond latencies while maintaining remarkable energy efficiency: features essential for mobile autonomous systems (Li Y. et al., 2021). Beyond these advantages, the intrinsic temporal dynamics of SNNs, coupled with biologically inspired plasticity mechanisms, such as spike-timing-dependent plasticity (STDP) (Białas and Mańdziuk, 2021), provide a natural substrate for implementing adaptive learning rules that operate continuously in real time. These properties make SNNs particularly well-suited for the development of controllers capable of learning directly from dynamic sensory-motor interactions, thereby offering a biologically grounded and computationally efficient pathway toward robust decision-making in complex environments (Chen et al., 2024).

Despite their considerable promise, the application of SNNs to address the exploration-exploitation dilemma remains at a relatively nascent stage. Many existing SNN-based controllers employ a single, generic STDP rule combined with fixed noise, yielding decision-making processes that lack adaptive modulation (Rahman and Yusoff, 2025). Even the most advanced three-factor SNN models, developed for demanding tasks such as quadruped locomotion, often continue to rely on hand-crafted exploration signals and fail to establish a clear actor-critic distinction (Schmidgall and Hays, 2023b). Although recent progress in neuromorphic reinforcement learning has extended SNNs to continuous control domains, these efforts reveal notable limitations: some introduce fully spiking actor networks yet remain dependent on surrogate-gradient backpropagation and non-spiking, floating-point critics (Kumar et al., 2025); others advance spike-based learning mechanisms but neglect explicit strategies for arbitration between exploration and exploitation (Zanatta et al., 2024). Collectively, these shortcomings underscore an evident gap in the field: the absence of a fully spiking architecture in which adaptive explore-exploit arbitration is governed by a unified and biologically plausible learning framework.

Neuroscience provides a functional blueprint for balancing exploitation and exploration. Distinct cortical-basal ganglia (BG) loops are believed to govern action selection, with a core BG pathway favoring the exploitation of known, valuable policies (Herz et al., 2022). In parallel, circuits involving the anterior cingulate cortex (ACC) and the subthalamic nucleus (STN) contribute to performance monitoring and generate behavioral variability, which is essential for exploration (Tervo et al., 2021; Laquitaine et al., 2024). Importantly, this process is dynamic rather than static, being modulated by neuromodulators such as acetylcholine (ACh) and dopamine (DA) (Chantranupong et al., 2023). Tonically active neurons (TANs) are thought to play a central role by signaling unexpected events and gating synaptic plasticity, thereby shaping both learning and action selection (Reynolds et al., 2022; Zhu et al., 2024).

**Figure 1:**
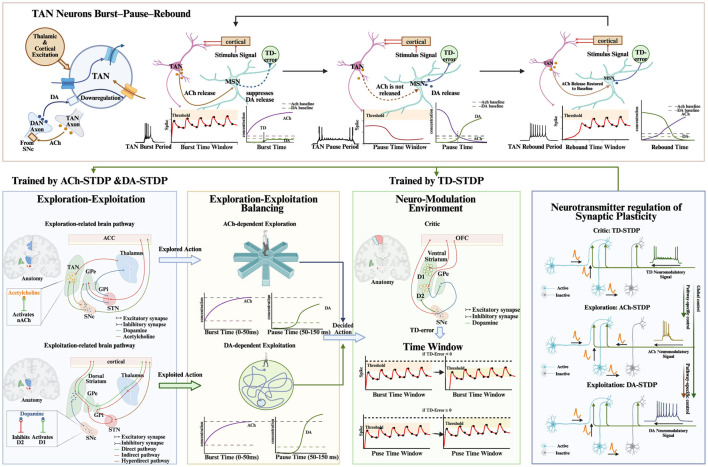
The adaptive explore-exploit balancing process in SpikeAEC, which integrates multiple core components into a unified architecture. Exploitative actions are generated by a basal ganglia (BG) model implementing Go/No-Go/Brake competition **(bottom-left)**, while exploratory actions arise from an ACC-GPe-STN circuit. Conflict between these pathways is resolved by a TAN-based Arbitrator, which operates through a “burst-pause-rebound” cycle triggered by the Critic's TD error **(top panel)**. This cycle regulates the release of neuromodulators, acetylcholine (ACh) and dopamine (DA), that gate a unified three-factor learning framework **(bottom-right)**. Within this framework, pathway-specific plasticity is achieved through TD-STDP for the Critic, DA-STDP for the Actor, and ACh-STDP for the Explorer, enabling targeted and functionally segregated adaptation.

Building on this division of labor, a proposed SpikeAEC controller operationalizes these principles within a fully spiking architecture (see Figure 1). Sensory input from event-based cameras, integrated with proprioceptive state variables, is transformed into spike trains and routed to two parallel subnetworks. The Actor, modeled after the BG, generates exploitative actions, whereas the Explorer, inspired by the ACC-STN pathway, produces adaptive exploratory behaviors. Central to this architecture is an Arbitrator module, implemented through a population of TANs, which resolves competition between the two pathways. The Arbitrator dynamically adjusts its firing propensity based on a global TD error signal computed by a Critic SNN. This modulation produces a characteristic TAN burst-pause cycle, yielding a transient biphasic neuromodulatory signal that simultaneously (a) probabilistically selects the executed action and (b) regulates pathway-specific plasticity across the network. Through this fully spiking, closed-loop design, SpikeAEC achieves an online, biologically grounded mechanism for maintaining an adaptive balance between exploration and exploitation.

The proposed AEC architecture makes three key contributions:

1. **Biological grounding:** To our best knowledge, SpikeAEC is the first fully spiking controller to integrate a BG-based Actor, an ACC-STN-inspired Explorer, and a TAN-based Arbitrator, enabling an online, neurally plausible balance between exploration and exploitation.
2. **Unified three-factor learning:** The Arbitrator's TAN cycle provides a unified neuromodulatory signal, where phasic ACh and DA, time-aligned with the global TD error, drive pathway-specific plasticity at each synapse without relying on backpropagation.
3. **Practical effectiveness:** In simulation, SpikeAEC outperforms several state-of-the-art brain-inspired baselines in convergence speed, trajectory efficiency, and cumulative reward.

Our motivation is to pioneer a new class of brain-inspired controllers that move beyond static, hand-crafted rules toward adaptive, online learning. By functionally embodying the brain's distinct mechanisms for exploitation, exploration, and arbitration within a SNN, SpikeAEC demonstrates a viable path to achieving robust and efficient autonomy. We believe this architectural blueprint, grounded in both neuroscientific principles and robotic practice, offers a scalable foundation for future intelligent systems, from agile drones to adaptive robotic manipulators.

## 2 Neuromorphic foundations

**Figure 2:**
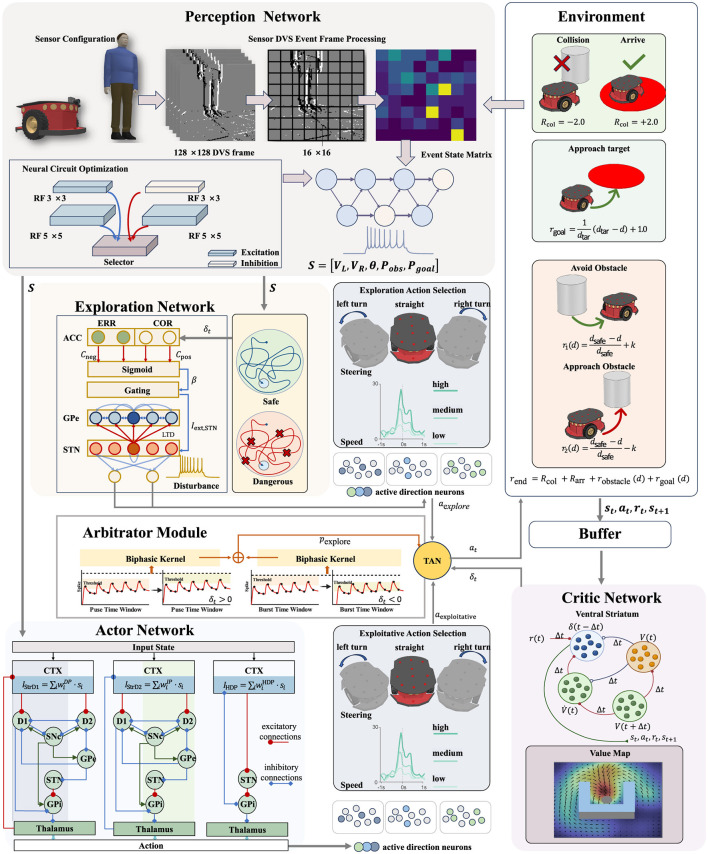
Overview of the SpikeAEC Architecture. A hybrid-trained SNN processes DVS events into a state vector *S*_*t*_. This state drives a BG-inspired Actor that proposes exploitative actions and an ACC-STN-inspired Explorer that generates exploratory ones. The Arbitrator resolves the conflict: a TD-error-modulated TAN signal determines the exploration probability *p*_explore_, and the final action *a*_*t*_ is selected via a probabilistic draw. Finally, the Critic computes the TD-error from the resulting transition, feeding it back to both arbitration and learning to complete the closed loop.

The SpikeAEC architecture, illustrated in Figure 2, is built upon key principles from computational neuroscience, including biologically plausible neuron models and neuromodulated synaptic plasticity. This section details these foundational components.

### 2.1 Biologically plausible neuron models

The foundation of our controller lies in SNNs, the third generation of neural networks that emulate the brain's event-driven, energy-efficient computational principles (Salaj et al., 2021). While traditional deep learning models face significant challenges with power consumption and latency on mobile robotic platforms (Higa et al., 2019), SNNs offer a promising path toward real-time, online learning and control.

A fundamental design consideration in any SNN is the choice of neuron model, as it determines both the computational expressiveness and the efficiency of the network. Existing models span a broad spectrum: at one extreme, the Leaky Integrate-and-Fire (LIF) neuron offers high computational efficiency but limited dynamical diversity (Karlsson and Kämäräinen, 2025); at the other, the Hodgkin-Huxley (HH) model provides a detailed biophysical representation at the cost of substantial computational overhead (Valle and Madureira, 2022). The Izhikevich model occupies a compelling middle ground, combining efficiency with biological realism. With only a small set of tunable parameters, it is capable of reproducing more than 20 empirically observed firing patterns, including regular spiking, intrinsic bursting, and chattering, thereby offering a versatile and computationally tractable framework for large-scale SNN simulations (Wang et al., 2022).

This versatility is particularly critical for constructing architectures that emulate the brain's functional specialization. Whereas many SNN applications in robotics rely on homogeneous neuron populations, our approach diverges by establishing neural diversity as a foundational design principle. The Izhikevich model is employed not merely as a generic computational unit but as a framework for instantiating heterogeneous neural populations with distinct functional roles. By tailoring electrophysiological properties to specific modules, the Actor (BG-like), the Explorer (ACC-STN-like), and the Arbitrator (TAN-like), we embed functional segregation directly into the network's structure. This design choice underpins the emergent dynamics of the SpikeAEC architecture, enabling it to replicate specialized neural processing and coordination observed in biological systems.

This design embodies a deliberate trade-off between biological plausibility and computational efficiency. While the Izhikevich model imposes a higher computational burden than the Leaky Integrate-and-Fire (LIF) model—owing to its coupled differential equations—this cost is mitigated through a hybrid architectural strategy. In our framework, high-dimensional visual input (Perception) is processed by LIF neurons, chosen for their simplicity and ability to support high-throughput computation. In contrast, the more computationally demanding Izhikevich neurons are selectively applied within the Controller, which operates on a considerably smaller state space. As the control network consists of fewer than 2,000 neurons—significantly fewer than those in the perception pipeline—the additional overhead introduced by the Izhikevich model remains negligible. This hybrid configuration enables SpikeAEC to function in real time while still benefiting from the richer neuronal dynamics essential for implementing the variable firing modes that underlie the system's exploration-exploitation behavior.

### 2.2 Neuromodulated three-factor learning

Beyond single-neuron dynamics, network-level adaptation in SNNs is driven by synaptic plasticity rules. Although STDP provides a foundational Hebbian learning mechanism (Białas and Mańdziuk, 2021), it is insufficient for goal-directed behavior because it lacks the capacity to solve the credit assignment problem, i.e., attributing global outcomes to local synaptic modifications (Gan and Qiao, 2025). To overcome this limitation, three-factor learning rules have emerged as a powerful and biologically plausible framework. Under this paradigm, the co-activation of pre- and post-synaptic neurons establishes a transient synaptic “flag,” referred to as an eligibility trace (Florian, 2007). A durable synaptic weight change occurs only if a third factor, a neuromodulatory signal conveying information about reward, novelty, or error, arrives during the lifetime of this trace. While the theoretical basis for this mechanism was proposed decades ago (Schmidgall and Hays, 2023a), conclusive experimental evidence for eligibility traces has only been established in recent years (Hong et al., 2022), thereby strengthening the biological foundation of this learning model.

Applications of this principle in robotics have generally followed two main directions. The first concerns low-level motor adaptation, in which the third factor encodes external physical properties of the environment. For instance, recent studies have employed neuromodulatory signals to represent terrain friction, enabling SNN controllers to adjust synaptic weights in real time to maintain stable locomotion (Yang et al., 2021). In such cases, neuromodulation primarily aligns the robot's motor dynamics with its physical surroundings.

A second, complementary line of work, within which our study is situated, applies neuromodulation to high-level cognitive control, particularly in resolving the exploration-exploitation dilemma. In this context, the third factor represents an internally generated evaluative signal rather than an external environmental state. Well-established insights from neuroscience provide guiding principles: DA encodes reward prediction errors that reinforce successful actions, while ACh signals uncertainty and promotes exploratory behavior (Krishnan et al., 2022). Despite their conceptual promise, translating these mechanisms into robust robotic controllers has proven challenging. Early implementations of reward-modulated plasticity, although effective in simple environments (He et al., 2021; Mozafari et al., 2019), were often restricted to shallow networks and low-dimensional tasks (Zhao et al., 2022). More advanced methods capable of training deep recurrent SNNs on complex benchmarks frequently depend on extensive task-specific manual tuning of network architectures and hyperparameters, thereby limiting scalability and generalization (Deng et al., 2023).

These persistent limitations, combined with the reliance on a single global reinforcement signal or hybrid non-spiking components, underscore a fundamental gap in the development of truly integrated cognitive architectures. To address this gap, we introduce SpikeAEC, a framework that leverages three-factor learning with internally generated, functionally distinct neuromodulators. By explicitly gating exploitation and exploration pathways within a fully spiking architecture, SpikeAEC provides a unified, biologically grounded solution for adaptive cognitive control.

## 3 Brain-inspired networks

### 3.1 Perception network

In Figure 2, a Dynamic Vision Sensor (DVS) captures visual input in the form of asynchronous events with microsecond-level temporal precision. These events are aggregated over 50 ms simulation windows, spatially downsampled using 16 × 16 binning, and then temporally stacked to form compact event frames. Each frame is subsequently encoded into Poisson-distributed spike trains with a maximum firing rate of 300 Hz, preserving critical timing information while substantially reducing the data volume. These spike trains are processed by a perception SNN that operates across multiple spatial scales to extract both fine-grained local features and broader global structure. Drawing inspiration from recent advances in neural circuit evolution (Li S. et al., 2021), the internal architecture of the perception SNN (Table 1) incorporates adaptive circuit motifs in which neuronal populations with heterogeneous receptive fields (RFs, 3 × 3 and 5 × 5) compete for synaptic connectivity to a centralized “Selector” population.

**Table 1:** Simulation parameters and network specifications.

**Module**	**Parameter/ component**	**Value/ specification**
Simulation	Time resolution	*dt* = 0.1 ms
	Integration window	*T*_sim_ = 50 ms
Perception	Input resolution	128 × 128 (DVS events)
	Preprocessing	16 × 16 (Poisson encoding)
	Conv. motifs (Local)	16 × 16 × 32 (LIF)
	Conv. motifs (Global)	16 × 16 × 32 (LIF)
	Feature selector	512 Neurons (LIF)
	LIF parameters	τ_*m*_ = 10.0 ms, τ_*syn*_ = 2.0 ms, *t*_*ref*_ = 2.0 ms
		*V*_*th*_ = −55.0 mV, *V*_*reset*_ = −70.0 mV
Control	Actor (D1/D2)	500 Neurons (Each)
	Explorer (GPe / STN)	200 Neurons (Each)
	Arbitrator (TAN)	100 Neurons
	Critic (Ventral Striatum)	100 Neurons
	Izhikevich (RS) Params	*a* = 0.02, *b* = 0.2, *c* = −65, *d* = 8, *v*_*peak*_ = 30mV
	Izhikevich (IB) Params	*a* = 0.02, *b* = 0.2, *c* = −55, *d* = 4, *v*_*peak*_ = 30mV
Learning & Neuromodulation	Learning rates	η_*C*_ = 0.012, η_*D*_ = 0.005, η_*E*_ = 0.002
	Burst learning rate	$\eta_{\text{burst}}=1.5\times 10^{-3}$
	Eligibility trace	τ_*e*_ = 1.5 s
	Neuromodulation kernel	*A*_ACh_ = 1.2, *A*_DA_ = 0.8
	Kernel time constants	τ_ACh_ = 25.0 ms, τ_DA_ = 120.0 ms
	Exploration scaling	*w*_*M*_ = 8.5, *b* = −2.4

Synaptic weights in the network are updated through a two-stage process. At the local level, an unsupervised Spike-Timing-Dependent Plasticity (STDP) rule modifies structural weights to eliminate redundant connections, giving rise to distinct connectivity motifs such as forward excitation and lateral inhibition. At the global level, a surrogate-gradient optimizer minimizes the mean-squared error between the network's predicted outputs and the corresponding ground-truth labels, which include both the obstacle probability *P*_obs_ and the simultaneously predicted goal probability *P*_goal_. The outputs of the SNN, together with time-stamped measurements of left and right wheel speeds (*V*_*L*_, *V*_*R*_), heading θ, along with the visual probabilities *P*_obs_ and *P*_goal_, collectively define the system's state vector: *S* = [*V*_*L*_, *V*_*R*_, θ, *P*_obs_, *P*_goal_]. This synchronized, multimodal representation provides the Actor, Explorer, and Critic modules with a richer and more context-aware input than visual information alone.

### 3.2 Izhikevich neuron model

The Izhikevich neuron model balances biophysical realism with computational efficiency, making it well-suited for replicating the diverse firing patterns observed in BG neurons (Izhikevich, 2003). For neuron *i* in population *X* and action channel *k*, the membrane potential *v*_*X, k, i*_ and recovery variable *u*_*X, k, i*_ evolve according to:


$$\begin{array}{l} \begin{array}{cc} \frac{\text{d}v_{X,k,i}}{\text{d}t} & =0.04v_{X,k,i}^{2}+5v_{X,k,i}+140-u_{X,k,i}+I_{X,k,i}^{\text{ext}}+I_{X,k,i}^{\text{syn}},\\ \frac{\text{d}u_{X,k,i}}{\text{d}t} & =a_{X}\left(b_{X}v_{X,k,i}-u_{X,k,i}\right). \end{array} \end{array}$$ (1)

A spike is registered when *v*_*X, k, i*_≥30mV, at which point the variables are reset as follows: *v*_*X, k, i*_←*c*_*X*_, *u*_*X, k, i*_←*u*_*X, k, i*_+*d*_*X*_. In this context, $I_{X,k,i}^{\text{ext}}$ represents the external drive, such as cortical or sensory input, while $I_{X,k,i}^{\text{syn}}$ denotes the total synaptic current received from the network. The parameters *a*_*X*_, *b*_*X*_, *c*_*X*_, and *d*_*X*_ are carefully selected for each neuron population to accurately replicate its unique electrophysiological characteristics (Izhikevich, 2007).

### 3.3 Basal-ganglia-inspired action network

As illustrated in Figure 2, the Actor network replicates the microcircuitry of the primate BG to enable effective action selection, with its internal state represented as a vector that encodes the nucleus-specific dynamics of its constituent neuronal populations:


$$X={\left[v_{\text{D1}},u_{\text{D1}},v_{\text{D2}},u_{\text{D2}},v_{\text{STN}},u_{\text{STN}},v_{\text{GPe}},u_{\text{GPe}},v_{\text{GPi}},u_{\text{GPi}}\right]}^{\text{T}}.$$ (2)

Each (*v, u*) pair follows the Izhikevich model, with parameters specifically tuned to replicate the electrophysiological signature of its corresponding nucleus, for example, bursting activity in the STN, tonic firing in the GPe/GPi, and regular spiking in the D1/D2 medium spiny neurons (MSNs). Spike trains generated by the Perception module serve as cortical input to the BG model, which processes this input through three parallel pathways that ultimately converge on the GPi, the output nucleus of the BG.

#### 3.3.1 Functional architecture and pathways

The direct pathway (Go) involves cortical excitation of D1-MSNs, which directly inhibits the GPi; this pathway is potentiated by high DA levels, thereby strongly facilitating action release (Zhang et al., 2023). In contrast, the indirect pathway (No-Go) begins with cortical excitation of D2-MSNs and triggers a multi-step loop through the striatum, GPe, STN, and finally the GPi, resulting in excitation of the GPi. Elevated DA levels suppress this pathway at its origin, the D2-MSN stage, weakening the “No-Go” signal (Zhu et al., 2023). Additionally, the Hyper-direct Pathway (Brake) involves monosynaptic projections from the cortex to the STN, whose widespread excitation of the GPi provides a rapid and global inhibitory control over motor output (Oswal et al., 2021).

#### 3.3.2 GPi dynamics and dopaminergic modulation

We model this complex competition using a single, functionally representative equation for the GPi neurons:


$$\begin{array}{l} \frac{\text{d}v_{\text{GPi},k,i}}{\text{d}t}=\left(0.04v_{\text{GPi},k,i}^{2}+5v_{\text{GPi},k,i}+140-u_{\text{GPi},k,i}\right)\\ \;\underset{\text{Direct, Go}}{\underset{︸}{-w_{\text{D1}\to \text{GPi}}c_{\text{D1}}(\text{DA})\sum_{j}S_{k,j}^{\text{D1}}}}\\ \;\underset{\text{Indirect, No-Go}}{\underset{︸}{+w_{\text{STN}\to \text{GPi}}c_{\text{D2}}(\text{DA})\sum_{j}S_{k,j}^{\text{STN}}}}\\ \;\underset{\text{Hyper-direct, Brake}}{\underset{︸}{+w_{\text{H}\to \text{GPi}}\sum_{l,j}S_{l,j}^{\text{STN},\text{H}}}},\\ \frac{\text{d}u_{\text{GPi},k,i}}{\text{d}t}=\;a_{\text{GPi}}(b_{\text{GPi}}v_{\text{GPi},k,i}-u_{\text{GPi},k,i}). \end{array}$$ (3)

In (3), the three synaptic input terms directly correspond to the “Go,” “No-Go,” and “Brake” pathways. The opposing effects of dopamine are explicitly incorporated via two gain functions, *c*_D1_(DA) and *c*_D2_(DA), which are modeled as opposing sigmoids. The “Go” term delivers channel-specific, DA-potentiated inhibition. Conversely, the “No-Go” term's influence is modulated by *c*_D2_(DA), which acts as a gain factor that decreases with rising DA levels, effectively capturing DA's net inhibitory influence on this excitatory pathway. The “Brake” pathway supplies a strong, DA-independent excitatory drive. Finally, the recovery dynamics ($\frac{du_{\text{GPi},k,i}}{dt}$) are tuned to produce tonic, adapting firing, representing a baseline level of motor inhibition.

#### 3.3.3 Exploitative action readout

Action selection is implemented through a winner-take-all mechanism that relies on GPi firing rates, which reflect the residual inhibition associated with each potential action. At discrete decision intervals, the average firing rate *f*_*k*_ for each channel *k* is calculated over a short sliding window:


$$\begin{array}{l} f_{k}=\frac{1}{nT}\overset{n}{\underset{i=1}{\sum }}\overset{t_{0}}{\underset{t=t_{0}-T}{\sum }}S_{\text{GPi},k,i}\left(t\right). \end{array}$$ (4)

The exploitative action *a*_exploitative_ is selected as the channel with the minimal firing rate, defined as *a*_exploitative_ = argmin_*k*_*f*_*k*_. This selected channel represents maximal disinhibition. The degree of disinhibition, reflected by how low *f*_*k*_ is, is then linearly mapped to the robot's wheel speeds, thereby directly linking the network's spiking dynamics to continuous motor commands. If all channels exhibit high, saturating firing rates, indicative of a strong “No-Go” or “Brake” state, the robot defaults to stopping or moving at minimal velocity.

### 3.4 ACC-GPe-STN-inspired exploration network

The Explorer network is responsible for generating complete, state-dependent exploratory actions. Unlike a simple perturbation mechanism, it operates as an independent expert that proposes an alternative action *a*_explore_, which competes with the Actor's exploitative proposal for control over behavior. This network's design draws inspiration from the functional roles of the ACC in performance monitoring and the GPe-STN loop in generating behavioral variability (Xiao et al., 2021).

#### 3.4.1 Cognitive-level gating (ACC-modulated vigilance)

At the cognitive level, an ACC-inspired module functions as a performance monitor by transforming the stream of reward prediction errors (δ_*t*_) from the Critic into a top-down control signal. This process involves tracking positive and negative prediction errors separately in two accumulator traces, *C*_pos_ and *C*_neg_:


$$\begin{array}{l} C_{\text{pos}}(t)=\gamma C_{\text{pos}}(t-1)+\max(0,\delta_{t}),\\ C_{\text{neg}}(t)=\gamma C_{\text{neg}}(t-1)+\max(0,-\delta_{t}). \end{array}$$ (5)

These traces, where γ∈[0, 1) is a decay factor controlling the memory of past performance, are integrated to compute a continuous vigilance index β^*^ as the normalized ratio of negative evidence:


$$\begin{array}{l} \beta^{*}\left(t\right)=\frac{C_{\text{neg}}\left(t\right)}{C_{\text{pos}}\left(t\right)+C_{\text{neg}}\left(t\right)+\epsilon }. \end{array}$$ (6)

The index β^*^ is then transformed into a global exploration gain, β, via a sigmoid function, where ϵ is a small constant to prevent division by zero. Crucially, within this architecture, the exploration gain β acts as a permissive gate for the entire Explorer network: when recent performance is poor (i.e., β is high), the gate opens, allowing the Explorer to generate alternative action proposals; conversely, when performance is good (i.e., β is low), the gate closes, and the Explorer remains silent, thereby avoiding interference with the Actor's successful policy.

#### 3.4.2 Neural-level action generator (GPe-STN loop)

At the core of the Explorer lies a fully spiking, recurrently connected GPe-STN network composed of Izhikevich neurons. The exploration gain β, originating from the ACC, directly regulates the network's excitability by modulating the total external input current, $I_{\text{STN}}^{\text{ext}}$, to the STN population:


$$\begin{array}{l} I_{\text{STN}}^{\text{ext}}\left(t\right)=\beta \left(t\right)\cdot \left(I_{\text{base}}+I_{\text{ctx}}\left(t\right)\right). \end{array}$$ (7)

In this formulation, *I*_base_ represents a baseline current, and *I*_ctx_(*t*) denotes the state-dependent input from the perception network. When β is low, the network receives minimal drive and remains quiescent; conversely, when β is high, the network becomes activated. The GPe-STN loop, whose dynamics are governed by Izhikevich equations (Wang et al., 2025), harnesses its rich chaotic activity to generate complex, bursting spike patterns within the STN population. This bursting activity, where *S*_STN, *k, i*_(*t*) denotes the spike train of the *i*-th STN neuron in channel *k*, is subsequently transformed into a well-formed action proposal, *a*_explore_, via a linear readout layer:


$$\begin{array}{l} a_{\text{explore}}\left(t\right)=\underset{k,i}{\sum }W_{k,i}^{\text{explore}}\left(t\right)\cdot S_{\text{STN},k,i}\left(t\right). \end{array}$$ (8)

The readout weights $W_{k,i}^{\text{explore}}$ are continuously adapted by a dedicated three-factor learning rule (see Section 3.6 for details), enabling the Explorer to identify which STN activity patterns yield effective exploratory actions over time.

### 3.5 TAN-mediated arbitration of exploitation and exploration

The final action *a*_*t*_ is selected through a competitive process between the Actor's exploitative proposal *a*_exploitative_ and the Explorer's exploratory proposal *a*_explore_. Rather than being resolved by a fixed rule, this Actor-Explorer competition is arbitrated online by a neuromodulatory mechanism inspired by the dynamics of TANs and their reciprocal interactions with ACh and DA (Bouabid et al., 2025). Our model proposes that the system learns when to explore by adaptively regulating the probability of initiating this arbitration process.

#### 3.5.1 The TD-modulated TAN burst threshold

At the core of the arbitrator lies a population of TANs, modeled using Izhikevich neurons with parameters specifically tuned to exhibit tonic baseline firing. In response to salient cortical inputs, these neurons can produce high-frequency bursts, which subsequently trigger a prolonged pause in their activity. We model this by making the TANs' burst threshold, *T*_burst_, adaptive, updating it after each decision outcome in accordance with the TD error δ_*t*_:


$$\begin{array}{l} T_{\text{burst}}\left(t+1\right)=T_{\text{burst}}\left(t\right)+\eta_{\text{burst}}\cdot \delta_{t}. \end{array}$$ (9)

In (9), η_burst_ denotes the learning rate. A TAN spike is registered as the onset of a burst only if its membrane potential *v*_*i*_ exceeds the dynamic threshold *T*_burst_. The membrane potential *v*_*i*_ corresponds to the *i*-th TAN neuron, whose dynamics are governed by the Izhikevich spiking neuron model (see Equation 1). This mechanism establishes a direct link between performance and the likelihood of initiating an exploratory arbitration cycle: a positive δ_*t*_ (indicating a better-than-expected outcome) raises the burst threshold, reducing the TANs' firing probability in response to subsequent stimuli. This suppresses new arbitration signals and favors continued exploitation of the Actor's successful policy. Conversely, a negative δ_*t*_ (reflecting a worse-than-expected outcome) lowers the threshold, increasing TAN sensitivity and the probability of a burst-pause cycle, thereby triggering a new arbitration signal and promoting exploration.

#### 3.5.2 Unified neuromodulatory signal

If a TAN burst is triggered (i.e., *v*_*i*_>*T*_burst_), the resulting firing pattern modulates the release of ACh and DA. We model this antagonistic interaction with a single, continuous neuromodulatory balance signal, *M*(*t*), which dynamically reflects the instantaneous ACh/DA ratio. During the TAN burst, ACh is released, driving *M*(*t*) toward a positive value (*M*>0), signaling uncertainty and the need to explore (Skirzewski et al., 2022). Conversely, during the subsequent TAN pause, the absence of ACh permits phasic DA release, pushing *M*(*t*) toward a negative value (*M* < 0), signaling reward-related evaluation and favoring reinforcement (Wang et al., 2014). This is implemented by filtering the spike train of each TAN neuron, *S*_TAN, *i*_(*t*), with a biphasic kernel *K*(*t*) and summing the results:


$$\begin{array}{l} M\left(t\right)=\underset{i}{\sum }K\left(t\right)*S_{\text{TAN},i}\left(t\right). \end{array}$$ (10)

Here, * denotes the convolution operator. The biphasic kernel *K*(*t*) is modeled as a difference of two exponential functions to capture the rapid ACh transient followed by the slower DA-related signal:


$$\begin{array}{l} K\left(t\right)=A_{\text{ACh}}e^{-t/\tau_{\text{ACh}}}-A_{\text{DA}}e^{-t/\tau_{\text{DA}}}, \end{array}$$ (11)

where *A*_ACh_ and *A*_DA_ are amplitude parameters, and τ_ACh_ < τ_DA_ are the time constants governing the decay rates of the ACh and DA components, respectively. This produces a sharp positive peak followed by a broader, shallower negative lobe. In the absence of a triggered burst, *M*(*t*) remains at a neutral baseline.

#### 3.5.3 Probabilistic action selection

The final action is selected probabilistically, with the likelihood of exploration determined by the neuromodulatory balance integrated over a decision window, *T*_decision_. First, this integrated signal, *M*_decision_, is computed:


$$\begin{array}{l} M_{\text{decision}}=\overset{T_{\text{decision}}}{\underset{0}{\int }}M\left(t\right)\text{d}t. \end{array}$$ (12)

This value is then transformed into the probability of choosing the Explorer's action, *p*_explore_, via the logistic sigmoid function σ:


$$\begin{array}{l} p_{\text{explore}}=\sigma \left(w_{M}\cdot M_{\text{decision}}+b\right), \end{array}$$ (13)

where *w*_*M*_ and *b* are fixed hyperparameters that scale and shift the neuromodulatory influence. Finally, the action *a*_*t*_ is sampled according to this probability:


$$a_{t}=\{\begin{array}{ll} a_{\text{explore}} & \text{if a random draw is less than}p_{\text{explore}},\\ a_{\text{exploitative}} & \text{otherwise}. \end{array}$$ (14)

This mechanism establishes a direct connection between learning, neurophysiology, and behavior. The TD error regulates the initiation of the arbitration cycle by modulating the burst threshold, thereby influencing the overall system balance. Once a cycle is triggered, the specific configuration of the cycle, determined by the ACh/DA balance, governs the real-time arbitration between the two expert policies. This results in a robust, biologically grounded closed-loop system that adaptively manages the trade-off between exploration and exploitation.

### 3.6 Three-factor neuro-modulation from critic network

**Figure 3:**
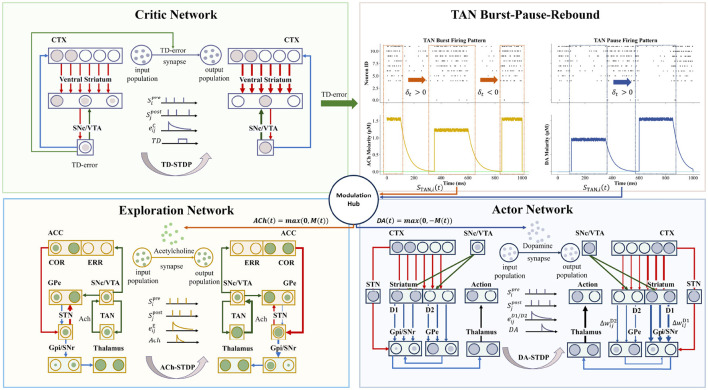
The Unified Three-Factor Learning Framework, detailing the biological motivation for pathway-specific plasticity. The Critic's temporal-difference (TD) error serves as the central driver of the learning process by modulating the activity of the Arbitrator's tonically active neurons (TANs). A negative TD error elicits a TAN burst, leading to the release of ACh to signal uncertainty, whereas a positive TD error induces a TAN pause, thereby facilitating DA release to signal reinforcement. These neuromodulators act as third factors that gate synaptic plasticity in a pathway-specific manner, enabling precise credit assignment. Specifically, the raw TD error modulates the Critic through a value-learning mechanism (TD-STDP); DA release gates plasticity within the Actor to reinforce policy updates (DA-STDP); and ACh release gates plasticity within the Explorer to refine exploratory strategies (ACh-STDP). Through this triadic modulation, the SpikeAEC framework achieves a biologically grounded and functionally segregated mechanism for adaptive learning.

The final component of our architecture is the Critic network, which assesses the outcomes of actions and generates the global learning signals that drive synaptic plasticity throughout the system. As illustrated in our proposed framework (Figure 3), this process is implemented via a unified, biologically plausible three-factor learning mechanism, wherein local synaptic eligibility traces are modulated by global neuromodulatory signals, a principle extensively examined in theoretical neuroscience (Van Hasselt et al., 2021).

#### 3.6.1 Critic, TD-error, and eligibility traces

The Critic is implemented as a SNN that learns the state-value function *V*(*s*). At each time step *t*, it receives the current state *s*_*t*_ and generates a corresponding value estimate, *V*(*s*_*t*_). Following the execution of action *a*_*t*_ and the receipt of reward *r*_*t*_, the Critic computes the global TD error, δ_*t*_, which serves as the primary learning signal driving all subsequent synaptic modifications and adaptive processes:


$$\begin{array}{l} \delta_{t}=r_{t}+\gamma V\left(s_{t+1}\right)-V\left(s_{t}\right). \end{array}$$ (15)

Concurrently, each plastic synapse within the Actor, Explorer, and Critic networks maintains a local eligibility trace, *e*_*ij*_. This trace captures recent pre- and post-synaptic spike coincidences, forming a short-term synaptic memory that identifies which connections were recently active and are thus “eligible” for modification. This mechanism is critical for bridging the temporal gap between neural activity and delayed feedback (Sarwat et al., 2022; Lee et al., 2024). The evolution of the eligibility trace is governed by the following rule:


$$\begin{array}{l} \frac{\text{d}e_{ij}}{\text{d}t}=\frac{1}{\tau_{e}}\left(-e_{ij}+S_{j}^{\text{pre}}\left(t\right)S_{i}^{\text{post}}\left(t\right)\right). \end{array}$$ (16)

Here, $S_{j}^{\text{pre}}\left(t\right)$ and $S_{i}^{\text{post}}\left(t\right)$ represent the presynaptic and postsynaptic spike trains respectively, τ_*e*_ denotes the decay constant, and the product term represents a Hebbian co-activation event. The eligibility trace plays a critical role in solving the synaptic-level credit assignment problem by identifying “who to blame” for a given outcome.

#### 3.6.2 Unified three-factor learning rules

All learning in the Actor and Explorer is driven by the neuromodulatory signals generated by the Arbitrator's TAN cycle, while the Critic learns directly from the raw TD error. As described previously, a triggered TAN burst-pause cycle produces a single neuromodulatory balance signal, *M*(*t*). This signal is then decomposed into distinct ACh and DA factors for gating plasticity in the Actor and Explorer. The positive and negative components of *M*(*t*) represent the phasic release of ACh and DA, respectively, such that ACh(*t*) = max(0, *M*(*t*)) and DA(*t*) = max(0, −*M*(*t*)). These two global signals are then broadcast to gate plasticity, effectively telling the synapses when and how to update their weights. This forms a canonical three-factor learning scheme: Δ*w*_*ij*_ = η·(Global Neuromodulator)·(Local Eligibility Trace).

##### 3.6.2.1 Critic learning (TD-STDP)

The Critic's cortico-striatal synaptic weights, $w_{ij}^{\text{Critic}}$, are updated to refine its value function estimates. In this context, the raw TD error δ_*t*_ serves as the third factor that gates the eligibility trace. This constitutes a direct implementation of TD learning within an SNN framework (Ma, 2023):


$$\begin{array}{l} \Delta w_{ij}^{\text{Critic}}=\eta_{C}\cdot \delta_{t}\cdot e_{ij}^{\text{C}}. \end{array}$$ (17)

##### 3.6.2.2 Actor learning (DA-STDP)

The Actor's cortico-striatal synaptic weights are modified based on the phasic DA signal, in alignment with its well-established role in reinforcement learning (Ryu et al., 2020). A positive DA signal, typically arising from a positive TD error, strengthens the direct pathway (Go) and weakens the indirect pathway (No-Go). Conversely, a low or absent DA signal produces the opposite effect:


$$\begin{array}{l} \begin{array}{cc} \Delta w_{ij}^{\text{D}1} & =\eta_{D}\cdot \text{DA}\left(t\right)\cdot e_{ij}^{\text{D}1},\\ \Delta w_{ij}^{\text{D}2} & =-\eta_{D}\cdot \text{DA}\left(t\right)\cdot e_{ij}^{\text{D}2}. \end{array} \end{array}$$ (18)

This is a true three-factor rule where DA provides the reinforcement context to the locally active synapses.

##### 3.6.2.3 Explorer learning (ACh-STDP)

The output weights of the Explorer module, $w_{ij}^{\text{Explorer}}$, are modulated by the phasic ACh signal. This mechanism reflects the established function of ACh in signaling uncertainty and enhancing behavioral flexibility by promoting the “unlearning” of previously reinforced but non-rewarding action policies. Through this process, the Explorer learns to identify and suppress patterns of STN activity that result in ineffective exploratory behavior:


$$\begin{array}{l} \Delta w_{ij}^{\text{Explorer}}=-\eta_{E}\cdot \text{ACh}\left(t\right)\cdot e_{ij}^{\text{E}}. \end{array}$$ (19)

Within this unified framework, local eligibility traces serve as synapse-specific memories, encoding “what just happened” at each connection. The global neuromodulatory signals, δ_*t*_, DA(*t*), and ACh(*t*), act as the essential third factor, providing contextual evaluations such as “was that good or bad?” and “should we explore or exploit?” This biologically grounded three-factor learning scheme enables the controller to simultaneously refine value estimates, reinforce successful motor behaviors, and enhance exploratory strategies, all within a single, coherent computational loop.

## 4 Experimental results

### 4.1 Experimental setting

**Figure 4:**
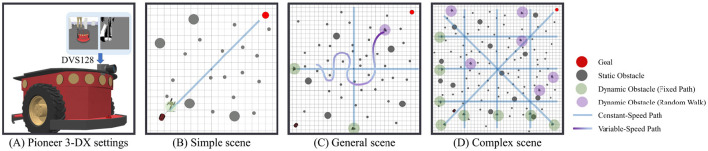
Experimental setup and evaluation arenas of increasing complexity. **(A)** The simulated Pioneer-3DX robot with its DVS camera. **(B)** Simple arena with sparse static obstacles and one predictable dynamic obstacle. **(C)** General arena with higher obstacle density and a mix of predictable and random-moving dynamic obstacles. **(D)** Complex arena with a dense field of static obstacles and numerous dynamic obstacles on both fixed and random paths, testing navigation in a cluttered environment.

All experiments were performed using the CoppeliaSim Pro simulator. The agent was a Pioneer 3-DX differential-drive robot, with a maximum linear velocity of *v*_max_ = 0.50m/s and a maximum angular velocity of ω_max_ = 1.6rad/s. The robot was equipped with a DVS128 event-based camera (Figure 4A) and was tasked with navigating from a fixed starting position to a designated target. The specific configuration details for each experimental arena are provided in Table 2.

**Table 2:** Parameters for experimental scenarios.

**Scenario**	**Arena size (m)**	**Obstacles (static/dyn.)**	**Dynamic speed (m/s)**
Simple	10 × 10	20/1	*v*_fixed_ = 0.5
General	15 × 15	50/3	*v*_fixed_ = 0.5; *v*_rand_(*k* = 0.15)
Complex	20 × 20	100/14	*v*_fixed_ = 0.5; *v*_rand_(*k* = 0.25)

The speed for random-path obstacles is *v*_rand_ = 0.5 × (1+*k*·ξ), where ξ is a random variable from a uniform distribution *U*[−1, 1]. The parameter *k* controls the magnitude of speed fluctuations.

We evaluated our model in three arenas of increasing complexity, as illustrated in Figures 4B–D. These environments include an escalating number of static obstacles and two types of dynamic obstacles: some that move along predefined paths at constant speed, and others that follow a random walk with variable speed. To facilitate the agent's learning process, we implemented a composite reward function that delivers dense feedback. This function comprises three components: (1) a reward for making progress toward the target, (2) a penalty or reward based on obstacle avoidance, and (3) a sparse terminal reward of +2.0 for successfully reaching the goal or −2.0 for colliding. The full formulation and parameter details of the reward function are provided in the caption of Figure 2.

### 4.2 Ablation study

#### 4.2.1 Functional validation of architectural components

As presented in Table 3, the full model (Row 1) serves as the performance benchmark, achieving the fastest convergence and most efficient navigation. When both the Explorer and the Arbitrator were ablated (Row 2), effectively reducing the architecture to a standard Actor-Critic baseline, performance degraded significantly, with the number of iterations to convergence (ITC) increasing by approximately 40%. Individual component ablations also led to marked declines: removing the Arbitrator TAN (Row 3) produced a less optimal final policy, reflected by a 21% increase in the average time after convergence (ATAC); disabling the Explorer's control ACC (Row 4) severely impaired learning efficiency, causing a 36% rise in the ITC; and ablating the Actor module BG (Row 6) confirmed the necessity of a dedicated exploitation pathway, as evidenced by an 18% increase in the ATAC. These results collectively highlight the complementary contributions of each architectural component to the system's overall performance.

**Table 3:** Ablation analysis of architectural modules.

**Configuration**	**Actor (BG)**	**Explorer (STN-GPe)**	**Explorer control (ACC)**	**Arbitrator (TAN)**	**ITC ↓**	**AT ↓**	**SDT ↓**	**MT ↓**	**ATAC ↓**
SpikeAEC (full model)	✓	✓	✓	✓	45.33 ± 0.45	66.14 ± 2.77	11.43 ± 1.35	90.99 ± 3.17	35.08 ± 1.48
w/o explorer & arbitrator^a^	✓	–	–	–	63.45 ± 2.01	83.70 ± 3.44	13.77 ± 1.11	109.66 ± 5.56	50.15 ± 2.45
w/o arbitrator (TAN)^b^	✓	✓	✓	–	56.87 ± 1.62	75.42 ± 2.88	12.88 ± 0.80	100.34 ± 5.01	42.37 ± 1.89
w/o explorer control (ACC)^c^	✓	✓	–	✓	61.67 ± 1.74	78.99 ± 3.56	14.00 ± 2.79	107.00 ± 6.67	49.29 ± 3.63
w/o explorer action (STN-GPe)	✓	–	✓	✓	53.12 ± 1.24	73.26 ± 3.05	12.61 ± 0.72	97.82 ± 4.50	39.92 ± 1.71
w/o actor (BG)	–	✓	✓	✓	51.44 ± 1.77	70.84 ± 2.64	11.97 ± 0.46	94.78 ± 4.73	41.50 ± 1.63
Random policy^d^	–	–	–	–	82.81 ± 2.30	102.08 ± 3.83	14.23 ± 2.15	130.53 ± 5.03	65.78 ± 2.73

“✓” indicates the module is active; “–” denotes it is ablated. Metrics: ITC, iterations-to-convergence; AT, average episode time (s); SDT, std. dev. of episode time (s); MT, max episode time (s); ATAC, average post-convergence episode time (s). All values are mean ± s.e.m. over 20 independent trials. Arrows (↓) indicate that lower values are better.

^*a*^This configuration serves as a standard Actor-Critic baseline, where the integrated exploration system is replaced by a simple ε-greedy strategy.

^*b*^Replaces dynamic, TD-error-based arbitration with a fixed probabilistic switch (e.g., ε = 0.1) between the Actor and Explorer policies.

^*c*^Disables the adaptive vigilance signal from ACC, causing the Explorer to generate unstructured random actions instead of a state-dependent policy.

^*d*^Represents a no-learning baseline where the agent selects actions randomly at each step.

#### 4.2.2 Functional hierarchy of three-factor learning rules

As shown in Table 4, the full model (Row 1) establishes the benchmark, achieving high path optimality (PO: 92.74%) and stable performance. The most significant degradation occurred when all learning pathways were reverted to a simple STDP rule (Row 7), which nearly collapsed the learning process, causing the average reward per goal (RSG) to drop by approximately 80%. Individual rule ablations also revealed critical dependencies on specific learning mechanisms. Replacing the Critic's TD-STDP with a simple STDP rule (Row 4) proved especially harmful, reducing PO by 36%, thereby underscoring the necessity of a precise TD-error signal for effective value estimation. Similarly, for the Actor, substituting DA-STDP with a generic STDP rule (Row 2) disrupted policy consolidation, resulting in a 39% increase in ATAC. Lastly, replacing the Explorer's ACh-STDP with simple STDP (Row 6) compromised the system's ability to prune inefficient exploratory behaviors, leading to a 15% decline in final PO. These findings highlight the essential role of specialized synaptic plasticity rules in supporting robust learning and adaptive behavior.

**Table 4:** Ablation analysis of neuromodulated three-factor learning rules.

**Configuration**	**DA-STDP actor**	**TD-STDP critic**	**ACh-STDP explorer**	**STDP actor**	**STDP critic**	**DA-STDP explorer**	**ITC ↓**	**PO ↑**	**ATAC ↓**	**LPS ↑**	**RSG ↑**
Full model	✓	✓	✓	–	–	–	44.67 ± 0.47	92.74 ± 0.06	35.04 ± 0.09	0.886 ± 0.008	8.535 ± 0.038
STDP for actor	–	✓	✓	✓	–	–	54.00 ± 3.06	84.77 ± 1.23	48.63 ± 6.44	0.702 ± 0.036	5.982 ± 0.197
DA-STDP for critic	✓	–	✓	–	✓	–	39.67 ± 2.49	76.32 ± 3.82	36.29 ± 3.99	0.608 ± 0.054	6.90 ± 5.00
STDP for critic	✓	–	✓	–	✓	–	72.33 ± 7.70	59.21 ± 5.95	59.86 ± 6.48	0.449 ± 0.098	4.31 ± 0.716
DA-STDP for explorer	✓	✓	–	–	–	✓	47.00 ± 3.20	89.02 ± 0.05	38.57 ± 0.06	0.783 ± 0.008	7.884 ± 0.039
STDP for explorer	✓	✓	–	–	–	✓	62.67 ± 7.05	79.28 ± 4.71	53.68 ± 6.10	0.617 ± 0.092	4.77 ± 0.755
All STDP	–	–	–	✓	✓	✓	84.33 ± 10.41	49.29 ± 9.74	83.22 ± 15.51	0.231 ± 0.096	1.69 ± 0.642

“✓” denotes the rule is active and “–” that it is ablated. Metrics: ITC↓ = iterations-to-convergence, PO↑ = path optimality as percentage of the shortest path, ATAC↓ = average episode time after convergence in seconds, LPS↑ = learning-phase stability measured as 1 - variance, RSG↑ = average reward per goal. All values are mean ± s.e.m. over 20 trials. Arrows indicate preferred direction.

### 4.3 Comparative study

#### 4.3.1 Comparison with state-of-the-art brain-inspired controllers

**Figure 5:**
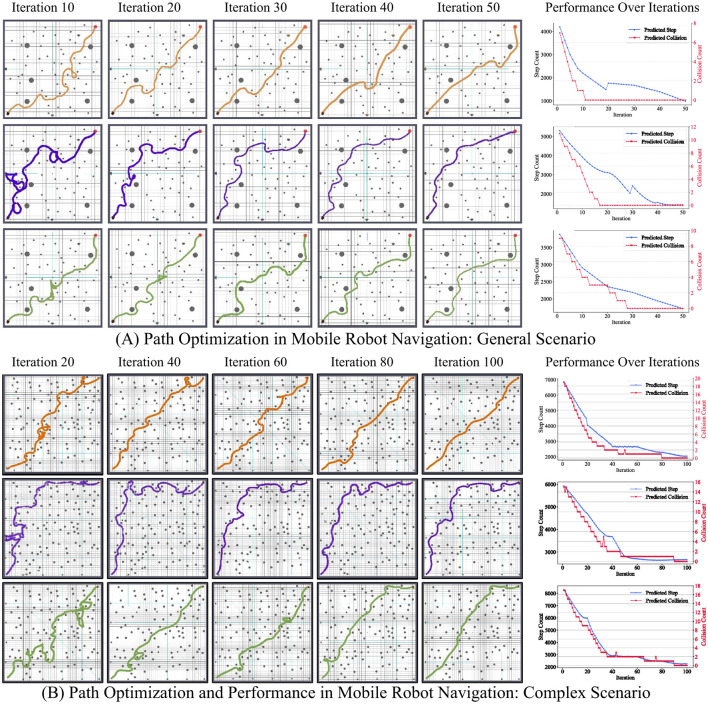
Trajectory evolution and learning curves under two arena complexities. **(A)** General arena with medium obstacle density. From top to bottom: SpikeAEC (orange), LiDAR-LK (purple), and BG-CB-SAC (green). Five snapshots (left → right) show the navigation path after 10, 20, 30, 40, and 50 training iterations. The plot on the right reports the mean episode length (blue, left axis) and cumulative collision count (red, right axis) versus iteration. **(B)** Complex arena with dense obstacle field. The layout is the same as in **(A)**; snapshots are taken at 20, 40, 60, 80, and 100 iterations. The accompanying curve illustrates the same performance metrics over 100 iterations.

As shown in Table 5 and Figure 5, SpikeAEC consistently outperforms a diverse set of representative brain-inspired controllers. Its performance gains are attributed to the synergistic integration of specialized sub-circuits that effectively overcome limitations present in alternative architectures. Hybrid models such as CBG-Hybrid (Wang et al., 2020) and BG-CB-SAC (Zhang et al., 2024a) incorporate cerebellum-like modules but rely on external supervision, which limits their efficiency. This is visually evident in Figure 5 (green trajectories), where BG-CB-SAC initially demonstrates inefficient, meandering paths and fails to discover the shortcut identified by SpikeAEC. As a result, CBG-Hybrid requires 24% more iterations to converge compared to SpikeAEC. Models like BG-Centric RL (Zeng et al., 2018) and CTX-Deep RL (Casanueva-Morato et al., 2023), which use unstructured noise for exploration, also struggle, leading to a 37% lower cumulative reward in BG-Centric RL. Earlier works like STN-GPe AE (Joseph et al., 2010) lack a dedicated critic and arbitrator, resulting in an 18% lower trajectory efficiency compared to SpikeAEC. Likewise, CTX-BG SNN (Wang et al., 2016) omits a neuromodulatory conflict resolution mechanism, leading to a 15% lower cumulative reward.

**Table 5:** Performance comparison between SpikeAEC and representative brain-inspired controllers.

**Model**	**ITC ↓**	**SDAS ↓**	**ANSE(5) ↓**	**ANSE(10) ↓**	**NSAC ↓**	**Reward ↑**
CBG-Hybrid (Wang et al., 2020)	67.59 ± 4.22	1107.24 ± 16.85	4,700.88 ± 37.22	3,954.31 ± 41.11	1651.92 ± 28.30	673.18 ± 72.44
BG-CB-SAC (Zhang et al., 2024a)	58.12 ± 3.67	1214.55 ± 11.48	5,137.67 ± 48.05	4,744.02 ± 55.63	2093.40 ± 20.01	869.53 ± 15.32
BG-Centric RL (Zeng et al., 2018)	71.88 ± 5.11	957.91 ± 9.27	5,184.13 ± 53.74	4,394.76 ± 40.03	1819.07 ± 31.67	580.64 ± 8.91
CTX-Deep RL (Casanueva-Morato et al., 2023)	96.04 ± 7.69	842.33 ± 18.47	5,508.49 ± 42.95	4,630.95 ± 49.92	2299.28 ± 12.22	224.81 ± 9.18
STN-GPe Sync (Mandali et al., 2015)	87.42 ± 6.23	1215.70 ± 13.74	4,435.21 ± 47.38	3,641.66 ± 42.07	1732.85 ± 11.66	309.47 ± 15.77
STN-GPe AE (Joseph et al., 2010)	77.73 ± 4.63	1040.18 ± 15.28	4,632.58 ± 40.51	3,827.43 ± 37.12	1900.75 ± 15.08	538.09 ± 18.52
CTX-BG SNN (Wang et al., 2016)	62.26 ± 4.05	900.61 ± 12.72	4,345.05 ± 30.06	3,640.89 ± 43.29	1798.36 ± 16.15	777.90 ± 34.25
Ours	54.71 ± 2.38	788.08 ± 7.63	3,900.27 ± 21.48	3,260.15 ± 25.94	1551.46 ± 18.09	915.15 ± 12.11

Arrows (↓: smaller is better; ↑: larger is better) indicate preferred direction. Metrics: ITC, iterations-to-convergence; SDAS, standard deviation of steps to accomplish the task; ANSE(k), average number of steps required for success over k repeated runs; NSAC, steps per episode after convergence; Reward, cumulative return. All values are reported as mean ± s.e.m. across 20 trials.

#### 4.3.2 Comparison with single-rule controllers

As shown in Table 6, methods relying on a single global reward signal, such as LiDAR-LK (Zhuang et al., 2023) and RSTDP-Track (Bing et al., 2019b), show limited performance. A global signal alone cannot provide the pathway-specific credit assignment needed for complex tasks. This is visually apparent in Figure 5 (purple trajectories), where the LiDAR-LK controller exhibits highly inefficient paths, even after convergence. Quantitatively, LiDAR-LK requires 19% more iterations to converge and achieves a 13% lower cumulative reward than SpikeAEC. Handcrafted supervision [Sup-RSTDP-Reach (Bing et al., 2019a)] or adaptive tuning (Auto-RSTDP-OA Lu et al., 2021) yields only marginal gains. Models emphasizing sensor-agnostic design or sparsity, such as Radar-CA (Van Damme et al., 2023) and Tiny-LK (Zhang et al., 2024b), also suffer in policy quality.

**Table 6:** Performance comparison of alternative learning-rule controllers.

**Model**	**ITC ↓ (mean ± sem)**	**SDAS ↓**	**ANSE(5) ↓**	**ANSE (10) ↓**	**NSAC ↓**	**Reward ↑**
LiDAR-LK (Zhuang et al., 2023)	110.33 ± 1.53	1,181.50 ± 33.05	5,722.60 ± 90.20	4,910.30 ± 64.40	2,570.31 ± 35.23	745.00 ± 5.01
RSTDP-Track (Bing et al., 2019b)	117.67 ± 2.53	1,258.91 ± 50.40	6,021.12 ± 95.06	5,149.21 ± 77.77	2,697.51 ± 42.46	713.33 ± 6.23
Radar-CA (Van Damme et al., 2023)	132.33 ± 2.51	1,455.71 ± 50.00	6,624.02 ± 103.66	5,677.27 ± 129.04	2,975.80 ± 59.38	565.00 ± 5.01
Sup-RSTDP-Reach (Bing et al., 2019a)	141.00 ± 3.61	1,608.18 ± 32.67	6,905.69 ± 90.39	5925.30 ± 62.29	3,097.03 ± 25.90	545.00 ± 5.01
RSTDP-LK (Bing et al., 2018)	147.33 ± 2.09	1,772.28 ± 32.90	7,083.71 ± 74.95	6,080.48 ± 64.19	3,194.29 ± 19.44	536.67 ± 6.23
Auto-RSTDP-OA (Lu et al., 2021)	152.33 ± 2.52	1,870.74 ± 33.05	7,183.87 ± 74.88	6,157.32 ± 47.59	3,269.22 ± 25.71	520.00 ± 4.99
Tiny-LK (Zhang et al., 2024b)	162.33 ± 2.53	2,034.84 ± 33.15	7,985.99 ± 74.40	6,822.34 ± 64.47	3,586.24 ± 35.02	676.67 ± 6.24
Ours	92.67 ± 2.52	951.78 ± 33.05	5,104.06 ± 74.95	4,358.88 ± 47.59	2,257.78 ± 25.79	853.33 ± 6.23

Arrows (↓: smaller is better; ↑: larger is better) indicate preferred direction. Metrics: ITC, iterations-to-convergence; SDAS, standard deviation of steps to accomplish the task; ANSE(k), average number of steps required for success over k repeated runs; NSAC, steps per episode after convergence; Reward, cumulative return. All values are reported as mean ± s.e.m. across 20 trials.

### 4.4 Brain-like consistency discussion

**Figure 6:**
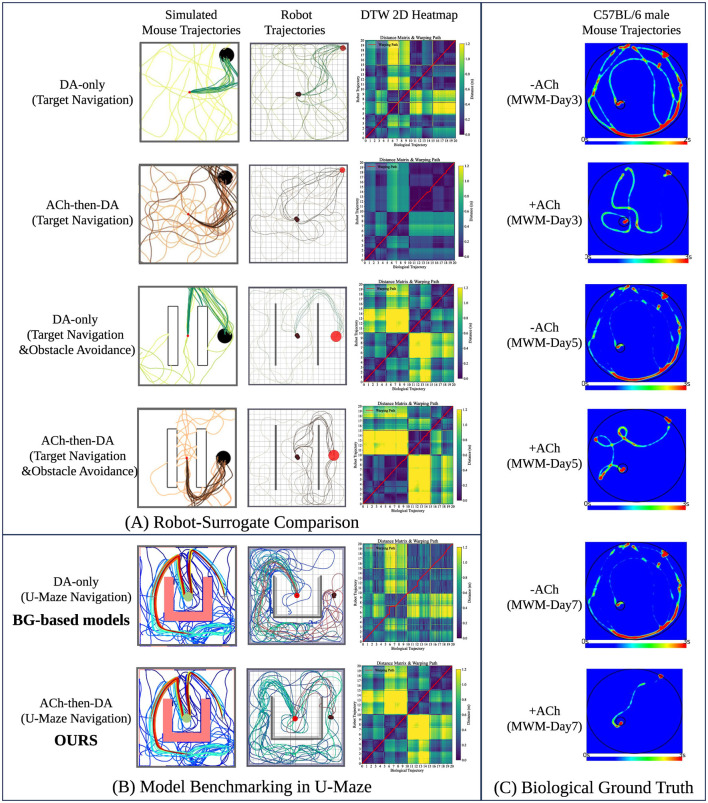
Neuro-behavioral alignment across scales: **(A)** Behavioral comparison between SpikeAEC and a simulated mouse surrogate. Top rows (DA-only): Absence of cholinergic modulation leads to perseverative circling and inefficient path integration, reflecting dynamics of dopamine-only learning. Bottom rows (SpikeAEC): Full ACh-then-DA sequence enables efficient pruning of suboptimal behaviors and promotes direct, goal-oriented navigation; **(B)** architectural validation in a U-maze task. Non-modular baseline (top) exhibits unstable switching between exploration and exploitation, whereas the modular SpikeAEC architecture (bottom) achieves consistent arbitration and stable behavioral transitions; **(C)** *in vivo* biological validation. Mice with intact cholinergic signaling (+ACh) demonstrate efficient navigational strategies, in contrast to the repetitive circling observed in animals with blocked ACh receptors (–ACh).

Figure 6A supports our central hypothesis that a sequential neuromodulation strategy—specifically, Acetylcholine (ACh) followed by Dopamine (DA)—confers significant advantages over a monolithic, dopamine-only signal. Under the DA-only condition (Rows 1 and 3), the agent displays “perseverative circling” behavior, repeatedly traversing suboptimal loops without consolidating a goal-directed trajectory. This inefficiency is quantitatively reflected in the Dynamic Time Warping (DTW) matrices, where elevated off-diagonal intensity (visible as yellow and green regions) indicates a poor alignment between the robot's path and the efficient biological reference. By contrast, the full ACh-then-DA sequence (Rows 2 and 4) implements a “prune-then-reinforce” mechanism: the ACh phase actively suppresses redundant exploratory behaviors, while the subsequent DA phase reinforces successful trajectories. This results in a sharp, linear diagonal in the DTW matrix, signifying strong behavioral alignment with biologically efficient navigation patterns.

Figure 6B illustrates the structural benefits of our modular controller design. The classical, non-modular Basal Ganglia (BG) controller (top row) exhibits limited capacity to arbitrate between exploration and exploitation. As reflected in its disorganized trajectory and noisy DTW matrix, the agent frequently oscillates between competing behavioral modes, failing to adopt a coherent strategy. In contrast, the SpikeAEC controller (bottom row), which incorporates functionally distinct Actor, Explorer, and Arbitrator modules, displays strong experimental consistency. The Arbitrator plays a critical role in gating transitions between behavioral states, enabling the system to rapidly converge on an optimal trajectory. This is evident from the clear, linear alignment observed in the corresponding DTW heatmap.

Figure 6C illustrates in vivo validation of the core computational principle underlying our model. In biological experiments, mice with pharmacologically blocked cholinergic receptors (–ACh group) exhibit persistent circling behavior, repeatedly failing to locate the platform—a pattern that closely parallels the “DA-only” failure mode observed in our system. In contrast, mice with intact cholinergic modulation (+ACh group, corresponding to our ACh-then-DA condition) transition rapidly from exploratory to goal-directed navigation. The structural similarity between the DTW profiles of the robot and the biological subjects underscores this cross-species behavioral alignment, providing compelling empirical support for the neuro-inspired design of the SpikeAEC architecture.

## 5 Conclusion

This paper introduced SpikeAEC, a fully spiking AEC controller that achieves robust real-time performance by emulating the brain's mechanistic division of labor for adaptive behavior. By integrating a TD-driven Arbitrator with pathway-specific, three-factor learning rules, SpikeAEC successfully balances exploration and exploitation in environments with sparse and delayed rewards. While demonstrated in navigation tasks, the architecture offers a general neuromorphic paradigm applicable to a broad range of autonomous systems. Future work will pursue three key directions: (1) porting the network to neuromorphic hardware platforms such as Loihi-2 and SpiNNaker-2 for on-board deployment; (2) extending the framework to continuous-action robotic manipulators; and (3) incorporating additional neuromodulators, such as serotonin, to address risk-sensitive decision-making and multi-goal behavior. We envision SpikeAEC not only as a practical, high-performance controller for real-world robotics, but also as a computational probe into the multi-scale biological principles that underlie intelligent and adaptive behavior.

## Data Availability

The raw data supporting the conclusions of this article will be made available by the authors, without undue reservation.
